# Single-cell RNA sequencing reveals the mediatory role of cancer-associated fibroblast *PTN* in hepatitis B virus cirrhosis-HCC progression

**DOI:** 10.1186/s13099-023-00554-z

**Published:** 2023-05-31

**Authors:** Chenhong Lin, Yeda Chen, Feng Zhang, Peng Zhu, Liangliang Yu, Wenbiao Chen

**Affiliations:** 1 Department of Endoscopy Center, Sir Run Run Shaw Hospital, Zhejiang University School of Medicine, Hangzhou, 310000 China; 2 Central Laboratory, People’s Hospital of Longhua, The Affiliated Hospital of Southern Medical University, Shenzhen, 518109 China; 3 Intensive Care Unit, The First Affiliated Hospital of Jinan University, Guangzhou, 510000 China; 4 Central Laboratory, Shenzhen Pingshan District People’s Hospital, Pingshan General Hospital, Southern Medical University, Shenzhen, 518110 China

**Keywords:** HBV, Cirrhosis, HCC, cancer-associated fibroblast, *PTN*, Single-cell RNA sequencing

## Abstract

**Background:**

Cancer-associated fibroblasts (CAFs) are essential stromal components in the tumor microenvironment of hepatocellular carcinoma (HCC). Hepatitis B virus (HBV) infection induces pathological changes such as liver fibrosis/cirrhosis and HCC. The aim of this research was to explore the novel mediators of CAFs to modulate HBV cirrhosis-HCC progression.

**Methods:**

The single-cell transcriptome data of HCC were divided into subsets, and the significant subset related to fibrotic cells, along with biological function, and clinical information of HCC was revealed by integrated data analyses. The cell communication, cells communicated weight analysis of signaling pathways, and key genes in signaling pathways analysis of significant CAFs subclasses were conducted to discover the novel gene of CAFs. Bioinformatics, *vitro* and HBV transfection assays were used to verify the novel gene is an important target for promoting the progression HBV cirrhosis-HCC progression.

**Results:**

Fibroblasts derived from HCC single-cell data could be separated into three cell subclasses (CAF0-2), of which CAF2 was associated with the HCC clinical information. Fibroblasts have opposite developmental trajectories to immune B cells and CD8 + T cells. CAF0-2 had strong interaction with B cells and CD8 + T cells, especially CAF2 had the highest interaction frequency and weight with B cells and CD8 + T cells. Moreover, PTN participated in CAF2-related pathways involved in the regulation of cell communication, and the interactions among CAF2 and PTN contributed the most to B cells and CD8 + T cells. Furthermore, the genes of *PTN*, *SDC1*, and *NCL* from PTN signaling were highest expression in CAF2, B cells, and CD8 + T cells, respectively, and the interaction of PTN- *SDC1* and *PTN*- *NCL* contributed most to the interaction of CAF2- B cells and CAF2-CD8 + T cells. Bioinformatics and vitro experiments confirm *PTN* was upregulated in HCC and promoted the proliferation of tumor cells, and HBV infection could initiate *PTN* to perform cirrhosis-HCC progression.

**Conclusion:**

Our findings revealed CAF was associated with hepatocarcinogenesis, and the functional importance of B cells and CD8 + T cells in modulating CAF in HCC. Importantly, *PTN* maybe a novel mediator of CAF to mediate HBV cirrhosis-HCC progression.

**Supplementary Information:**

The online version contains supplementary material available at 10.1186/s13099-023-00554-z.

## Introduction

Hepatocellular carcinoma (HCC) is one of the most common malignant tumors, which accounts for 70-90% of all primary liver cancer cases worldwide [1]. Risk factors for HCC vary by race and environment, and in Southeast Asia, about 80% of HCC cases are caused by hepatitis B virus (HBV) infection [2]. Although intensive hepatitis B vaccination efforts and the use of nucleoside antiviral drugs in this region have contributed to the prevention of HCC, for most patients, diagnosis is made at the middle or late disease stage when progression from chronic HBV infection to HCC occurs, resulting in poor prognosis [3]. The treatment of HCC includes surgery, chemotherapy, targeted drugs, liver transplantation, intervention and et al. Current clinical guidelines recommend comprehensive intervention, among which early diagnosis, early detection and early surgical resection are the key to improve the long-term therapeutic effect of HCC [4]. Therefore, the research of early diagnosis biomarkers of HCC, especially the disease caused by HBVs infection, is of great significance for improving the clinical management of HCC.

Chronic HBV infection progresses to HCC via an important intermediate pathological state, liver fibrosis/cirrhosis [5]. More than 80% of HCC occurs in fibrotic or cirrhotic livers, suggesting that liver fibrosis creates a precancerous environment in the liver, which promote HCC development [5, 6]. Fibroblasts, as essential stromal components of HCC, play an important role in the initiation, accumulation, and progression of liver fibrosis [6]. In addition, in HCC, fibroblasts can be transformed into cancer-associated fibroblasts (CAFs), a distinctive feature of the tumor microenvironment (TME), which has been extensively reported to modulate HCC progression [7]. As important stromal components of TME, CAFs mediate tumor-extracellular matrix communication, and are involved in extravascular formation, immune escape, and exosome molecule secretion outside tumors [7]. As a result, CAFs have been investigated for their potential role in HCC pathogenesis and as targets in HCC treatment [8]. Thus, a better understanding of the unique pathological functions of CAFs in the progression of HCC will provide a basis for development of precise therapy of HCC.

Pathologically, CAFs are considered to be highly heterogeneous: genetic changes and dysregulation of gene expression in normal fibroblasts generate CAFs [9]. Immunologically, CAFs interact with the TME network inside tumors in two ways: CAFs recruit immune cells, such as neutrophils and dendritic cells to promote immune escape and inhibit immune cells, such as B cells and T cells, to achieve immune tolerance [10]. Currently, single cell sequencing is valuable technology for investigating the communication between cancer cells and tumor microenvironment represented by CAFs, especially CAFs’ role in gene expression and immune cell regulation [11]. Here, using systematic data analysis, we accurately screened HCC for CAFs and grouped them into subclasses to evaluate their biological function, developmental trajectory and clinical correlation. Cell communication, signal pathway weight and key gene analysis, and in vitro experiments revealed the role of *PTN* expressed in CAFs in HCC, and the regulatory role of B cells and CD8 + T cells in *PTN*-mediated HBV cirrhosis-HCC progression.

## Methods

### Data preparation and preprocessing

Single-cell sequencing data (GSE125449 and GSE151530) in NCBI database were downloaded from GEO, including the expression matrices of the corresponding clinical features. GSE125449 dataset contained 12 HCC and 7 intrahepatic cholangiocarcinoma cases but only the 12 HCC sample data were retained. GSE151530 dataset consisted of 49 HCC and intrahepatic cholangiocarcinoma cases, but only 13 HCC samples were retained as independent validation data sets. The bulk of RNA sequence data from GEO including GSE63898 (168 cirrhotic and 228 HCC), GSE135251 (10 normal and 206 non-alcoholic fatty liver disease (NAFLD)), and GSE14520 (220 normal and 225 HCC) were used for clinical analyses. RNA sequencing data and corresponding clinical information of HCC were downloaded from TCGA database. In total, 365 samples with prognostic information were finally retained for subsequent analysis after removing the missing survival time and metastatic samples. This study was approved by the Ethics Committee of Shenzhen Longhua Hospital.

### Cell cluster analysis

The single cell sequencing data were subjected to standard downstream processing using Seurat R package. Briefly, single-cell sequencing data were filtered based on each gene being expressed in at least five cells and each cell expressing at least 500 genes. Next, the proportion of mitochondria and rRNA was calculated using the PercentageFeatureSet function, while ensuring that the genes expressed in each cell was greater than 500 and the mitochondrial content was less than 30%. After combining different samples, LogNormalize method was used for data normalization. Then, variable features were identified by normalizing integrated data using variance stabilization transformation. Subsequently, we used FindVariableFeatures methods to find highly variable genes. The ScaleData function was used to scale all genes and principal component analysis (PCA) was conducted to reduce the dimension to find anchors. Following, we selected the first 18 principal components and used FindNeighbors and FindClusters functions to cluster cells (set resolution = 0.2), which yielded 12 subsets. We selected the first 18 principal components and used clustering of UMAP to further reduce the dimension to display these 12 subsets.

### Cell type annotation and trajectory analysis

Annotation of cell subsets was performed using the Single R package, which annotates cell types using cell samples with known type labels by comparing unknown data sets with reference sets for similarity. For the annotation, we used two reference sets: HumanPrimaryCellAtlas Data and DatabaseImmuneCellExpression for annotation of all cell groups and fine annotation of the T cells subgroup, respectively. For trajectory analysis, Monocle R package was used to predict the development trajectory of each subpopulation, and the pseudotime of each cell was evaluated to measure the degree of cell differentiation. Branches of cell trajectories often arise because cells exhibit gene expression patterns. Cell trajectory branching is often due to differential gene expression patterns between cells. In the process of development, when a cell makes a fate choice, the evolution of the trajectory will branch. Consequently, one developmental lineage advances along one path, whereas the other lineage generates a second path. To accurately infer cell trajectories, only marker genes with an average expression value > 0.1 were retained and cells that expressed fewer than 200 genes were eliminated.

### Cell communication analysis

To study the role of various cell communication networks in the normal physiological proceses of the body, CellChat R package was used to systematically analyze cellular communication network. CellChat models the cell communication probability by integrating the prior knowledge of the expression of signal ligand, receptor and cofactor genes and their interactions [12]. “Secreted Signaling” from the CellChat database was selected as the reference data, and only signaling pathways with more than 10 cell interactions in both subclasses were retained.

### In vitro experiment

The human liver tumor cell lines HBV-positive Hep3B and HBV-negative Huh7 used in this study were obtained from Zhejiang University. These cells were cultured in an incubator at 5% CO_2_ and 37 °C. The cell medium was RPMI 1640 containing 10% fetal bovine serum. For real-time quantitative PCR (qPCR) analysis, total RNA was isolated from cells or tissues using TRIzol reagent (Life Technologies) according to the manufacturer’s instructions. mRNA was reversed transcribed into cDNA using All-in-One cDNA Synthesis Supermix Complete with a one-step reverse transcription reagent (Bimake, USA). The mRNA expression of the *PTN* was quantified by qPCR using SYBR Green Premix (YEASEN, China) on the 7500 Sequence Detection System (Applied Biosystems, China). 2^-ΔΔCT^ was used to calculate the relative gene expression, with GAPDH as the internal control for qPCR normalization. The primer sequences for these genes are listed in Table S1. For Western blot (WB) analysis, cells were lysed using RIPA buffer (Beyotime) at 4 °C directly. Total cellular protein concentrations were quantified with the BCA kit. Proteins were separated on 10% SDS-PAGE and then transferred to PVDF membranes (Bio-Rad, Hercules, USA). Then, the membrane was blocked with 5% skim milk and incubated at 4 °C overnight with the specific primary antibodies PTN antibody (1:300) and GAPDH primary antibody (1:500) and then with secondary antibodies. The chemiluminescent signal was detected and visualized using the enhanced chemiluminescence solution (ECL, Affinity). For cell proliferation analysis, the Cell Counting Kit-8 (CCK-8) was used to determine cell viability after 48 h according to the manufacturer’s instructions. Cell viability was calculated as the ratio of the treated to untreated cells. Cell migration was performed using transwell assays. Briefly, about 10^5^ cells were plated in DMEM supplemented with 10% FBS in the lower chamber of the transwell. The cells were stained with Giemsa staining solution and the invasive cells were counted under a microscope after incubation for 48 h. A wound healing assay was conducted to determine the migration ability of cells as previously described [13]. The plasmid pcDNA3.1-*PTN* used for overexpression of *PTN* was synthesized from TSINGKE (Tsingke Biotechnology, China). HCC cells were transfected plasmid pcDNA3.1-*PTN* or empty plasmid using Lipofectamine^TM3000^ transfection reagent. The transfection efficiency was then verified with qPCR and WB. Transfection of HBV DNA transient was performed by introducing 1.3mer HBV DNA (pHBV1.3) into Huh7 cells. EdU (5-ethynyl-20-deoxyuridine, Invitrogen) was used to stain nuclei, followed by flow cytometry (FCM) and immunofluorescence (IF) staining to detect cell proliferation as previously described [14].

### Statistical analysis

The K-means method was used to cluster all samples. Overall survival and progression-free survival were analyzed using Kaplan-Meier plot and log-rank *P* values were used to evaluate the difference in survival among the clusters. Univariate Cox proportion of hazard risk regression model was performed to calculate univariate risk ratio. Two independent variables were compared using Wilcoxon’s Sign Rank whereas nonparametric tests for three or more sets of data were performed using Kruskal–Wallis tests. All the statistical analyses and visualization were performed using R package (software v3.6.3) and GraphPad Prism 8.0 (San Diego, CA, USA).*P* < 0.05 was considered statistically significant.

## Results

### Fibroblast clustering identification based on single-cell RNA-seq profiling

**Fig. 1:**
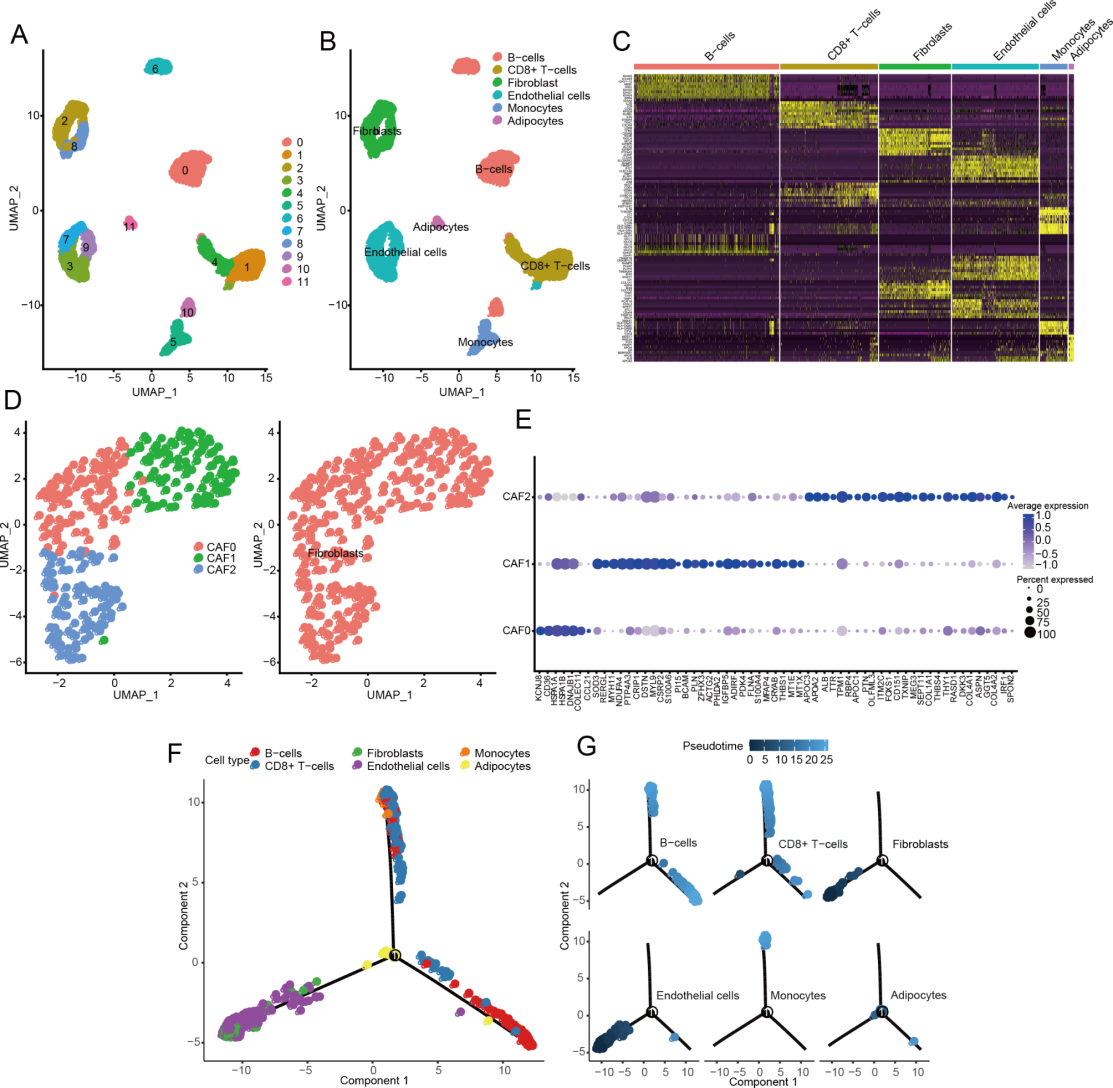
The subclass of cancer-associated fibroblast cells was obtained through analysis of HCC cell subset based on single-cell transcriptome data. (**A**) Cells were clustered into various types using the two-dimensional UMAP projection, each color represents the annotated phenotype of each types, (**B**) UMAP showing the categorization of 12 cell types into six cell subsets, including subset 2 and 8 which were designated as fibroblasts. (**C**) Heatmap showing 10% of marker genes with significant differences among 6 subsets. (**D**) The subdivision of cancer-associated fibroblast cells. (left) The UMAP showed the clustering results of fibroblast cells (3 subclasses, namely CAF0-2), and (right) the results of the notes of 3 fibroblast cells. (**E**) Dot plot showing expression level of marker genes in the 3 subclasses of fibroblast cells. (**F**) The trajectory analysis of six cell subsets. (**G**) The pseudotime analysis of six cell subsets

The single-cell RNA-seq profiling of GSE125449 dataset was used to group the cells. In PCA analysis, the appropriate number of principal components was evaluated for further dimensionality reduction of cell groups. Herein, we selected the top 18 PCAs for cell clustering (Figure S1), and all the cells could be classified into 12 cell types (Fig. 1A). Then, the 12 cell types were annotated to six cell subsets, including subsets 2 and 8 which were annotated as fibroblast (Fig. 1B). The six-cell subsets contained 2009 marker genes, including 213 and 216 genes in subsets 2 and 8, respectively. Furthermore, we performed differential expression analysis on marker genes across cell subsets and represented the top 10% differentially expressed marker genes in each subset on the heatmap (Fig. 1C). There were significant differences in marker gene expression across cell subsets, indicating that these marker genes are involved in the mechanism regulating cell differentiation.

The fibroblasts in subsets 2 and 8 were subdivided into 3 subclasses, namely CAF0-2 (Fig. 1D). All 3 subclasses were annotated as fibroblast (Fig. 1D), indicating the accuracy of cell sorting by single-cell RNA-seq profiling. The 3 subclasses comprised 59 marker genes, including 7, 26, 26 marker genes in CAF0, CAF1, and CAF2, respectively (Fig. 1E). As each of the six cell subsets (B cells, CD8 + T cells, fibroblasts, endothelial cells, monocytes, and adipocytes) performs a different function, we described the cell trajectory of CAF cell subsets. Cell trajectory branching occurs because cells exhibit different gene expression patterns and perform different biological functions. Further analysis showed that the differentiation trajectories of the six cell subsets were remarkably different, with fibroblasts and endothelial cells forming a distinct branch from B cells and CD8 + T cells (Fig. 1F). In addition, the pseudotime of fibroblasts and endothelial cells was earlier than that of B cells and CD8 + T cells (Fig. 1G), implying that fibroblasts and endothelial cells may be present in the very early stage of HCC and play an important function in HCC.

Meanwhile, the GSE151530 dataset was used for validation with similar analytic methods. By selecting the top 17 PCAs (Figure S2A, B) for cell clustering, we obtained seven cell subsets, including epithelial cells and six-cell subsets in the GSE125449 dataset (Figure S2C). Similar to GSE125449, the fibroblasts of GSE151530 can be further divided into 3 subclasses (Figure S2D), with 23, 50, 115 marker genes for sub-cluster0, sub-cluster1, and sub-cluster2, respectively. The top 10% differentially expressed marker genes in the 3 subclasses are represented in Figure S2E. Subsequently, by comparing the marker genes of GSE151530 with those of GSE125449 datasets, we found the three CAF subclasses in GSE125449 dataset with the majority of overlapping genes with GSE151530 (Figure S2F-H). These results demonstrated the accuracy of CAF mining by single-cell transcriptome data and the important functions of CAF cell subsets and their marker genes in investigating HCC.

### Association analysis between CAF0-2 and clinical information

**Fig. 2:**
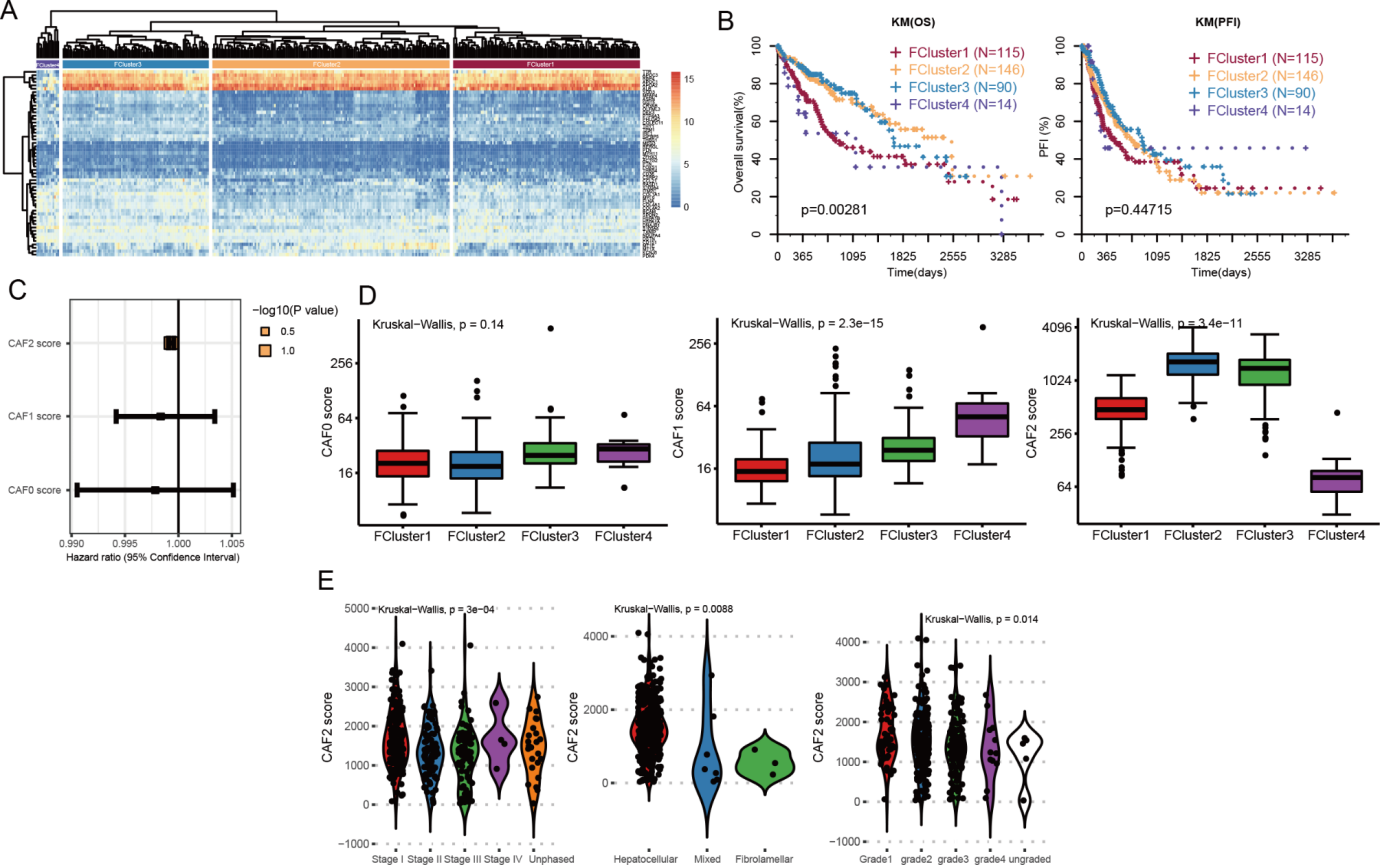
Three subclasses of CAFs associated with clinical information. (**A**) Hierarchical clustering of four distinct clusters (FCluster1-4) based on 57 marker genes from three subclasses (CAF0-2). (**B**) Kaplan–Meier plots showing the overall survival and progression-free survival among the patients. (**C**) Hazard ratio of CAF score was evaluated through the cox Hazard Scale Model. (**D**) The distribution of CAF0, CAF1 and CAF2 scores among the four distinct clusters (FCluster1-4). (**E**) Association of CAF2 with the HCC pathological stage, tissue samples, and pathological grade. Mixed: mixed sample

Six cell subsets were determined from single-cell RNA-seq profiling, including cells of the fibroblast type (CAF0-2). Three subclasses (CAF0-2) of marker genes of CAF cells were proposed to study the relationship between CAF and the prognosis of HCC using the TCGA HCC dataset. The expression profiles of 59 marker genes were determined to separate TCGA HCC samples into four distinct clusters (FCluster1-4) (Fig. 2A). Importantly, the overall prognostic survival of the four clusters showed significant differences, with worst prognosis for FCluster1, and FCluster2 and 3 had the best prognosis (Fig. 2B).

According to the marker genes of CAF0, CAF1 and CAF2 identified on the GSE125449 single-cell transcriptome, we evaluated the scores of TCGA HCC samples in these three subclasses, and took the average expression value of their marker genes as the CAF score in each HCC sample. Cox proportional hazards model was used to evaluate the relationship between CAF0-2 scores and overall survival. The results showed that the hazard ratios of CAF0-2 scores were all less than 1, indicating that they may be good prognostic factors, but only CAF2 had statistical significance (*p* = 0.033) (Figure C). Subsequently, we compared the CAF0-2 scores among the FCluster1-4, and found no significant difference between the score of CAF0 and those of the four groups of samples (*p* = 0.14). CAF1 score showed a gradual increase in FCluster1-4. CAF2 score for FCluster1 and 4 with poor prognosis was low but high in FCluster2 and 3 with good prognosis (Fig. 2D). In addition, CAF2 score was negatively correlated with pathological stage and pathological grade, and CAF2 score was highest in hepatocellular sample and lowest in mixed sample (Fig. 2E). However, we could not reveal any association between CAF2 score and tissue type, age, and sex of HCC (Figure S3). For CAF0 and 1, no significant relationship was observed between CAF score and clinical information (Figure S4A-L). These results indicated that CAF2 was an effective indicator for exploring the role of CAF in HCC.

### Analysis of communication between six cell subsets

**Fig. 3:**
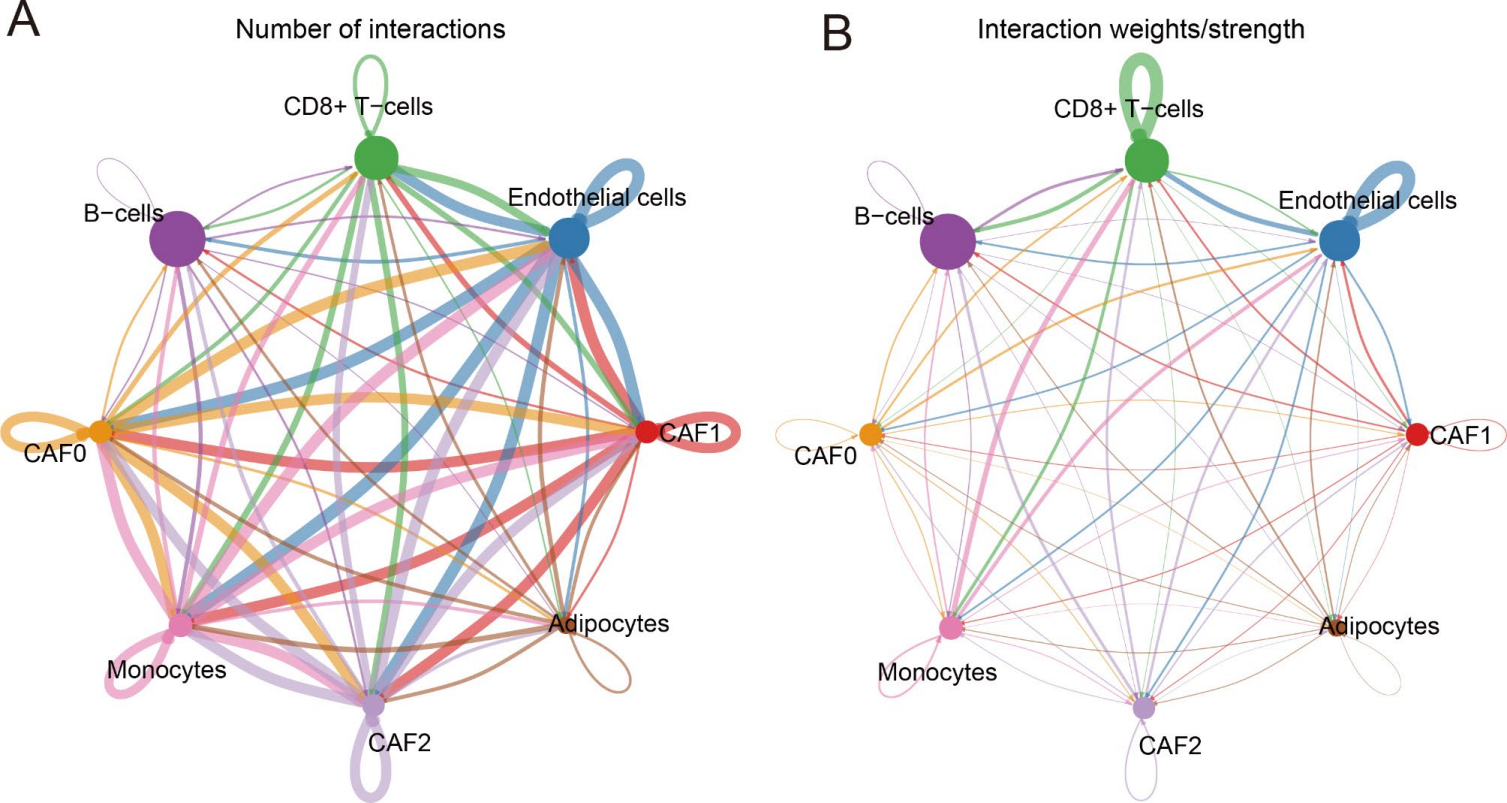
Communication links among the six cell subsets. The network of (A) frequency of interaction and weight intensity for the interaction among the six cell subsets

To obtain the interaction between CAF and other cell subsets, we analyzed the possible communication links between CAF0-2 and different cell subsets. CAF2 had the maximum interaction frequency with CAF0, CAF1, and endothelial cells, and some frequency communication with B cells and CD8 + T cells (Fig. 3A). Meanwhile, CAF2 had the highest weight intensity of interaction with B cells and CD8 + T cells (Fig. 3B). Moreover, we analyzed how each cell subset interacted with other cell populations. The results revealed that each of these cell subsets was associated with B cells and CD8 + T cells (Figure S5), especially CAF0-2 showed high frequency of interaction with B cells, CD8 + T cells, and endothelial cells (Figure S5A, E, G). Considering that B cells and CD8 + T cells are immune cells, CAF may be involved in the remodeling of the immune microenvironment.

### CAF2 communicated weight analysis of signaling pathways

**Fig. 4:**
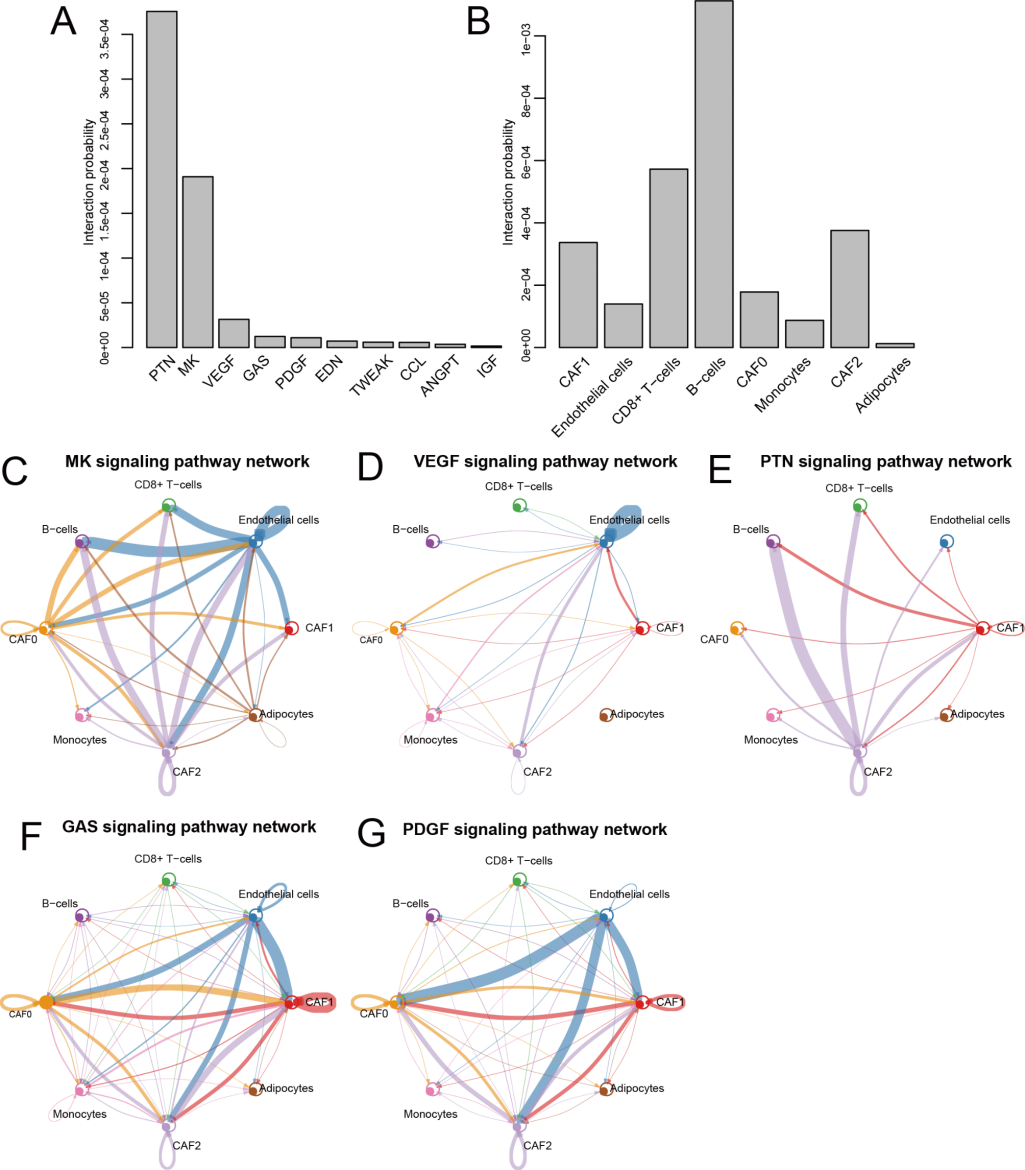
Analysis of CAF2-related signaling pathways. (**A**) The top 10 signaling pathways associated with CAF2. (**B**) The 10 signaling pathways linked to the interactions among six cell subsets. (**C-G**) The network of contribution weight of the top 5 CAF2-related signaling pathways (MK, VEGF, PTN signaling, GAS, PDGF) to the communication among six cell subsets

Since CAF2 was identified as a significant factor in HCC, we intensively investigated the signaling pathways that contribute to CAF2 communication. The top 10 signaling pathways with the highest interaction probability and the most contribution to CAF2 communication were PTN, followed by MK, VEGF, GAS, PDGF, EDN, TWEAK, CCL, ANGPT and IGF (Fig. 4A). The interactions among six cell subsets which contributed the most to signaling pathways were CAF2, B cells, and CD8 + T cells (Fig. 4B). Subsequently, we further concentrated on the top five CAF2-related signaling pathways with the largest contribution. The results showed that CAF2 was more or less associated with B cells and CD8 + T cells in all CAF2-related signaling pathways (Fig. 4C-G). Particularly, communication between CAF2 and B cells had the highest weight, followed by the communication between CAF2 and CD8 + T cells to the contribution of PTN signaling pathways (Fig. 4E). These results suggested that PTN mediated the effects of CAF2 -on cancer-associated fibroblast in HCC, and the interaction between CAF2-B cells and CAF2-CD8 + T cells may significantly regulate the mediation of *PTN*.

### Analysis of key marker genes in signaling pathways

**Fig. 5:**
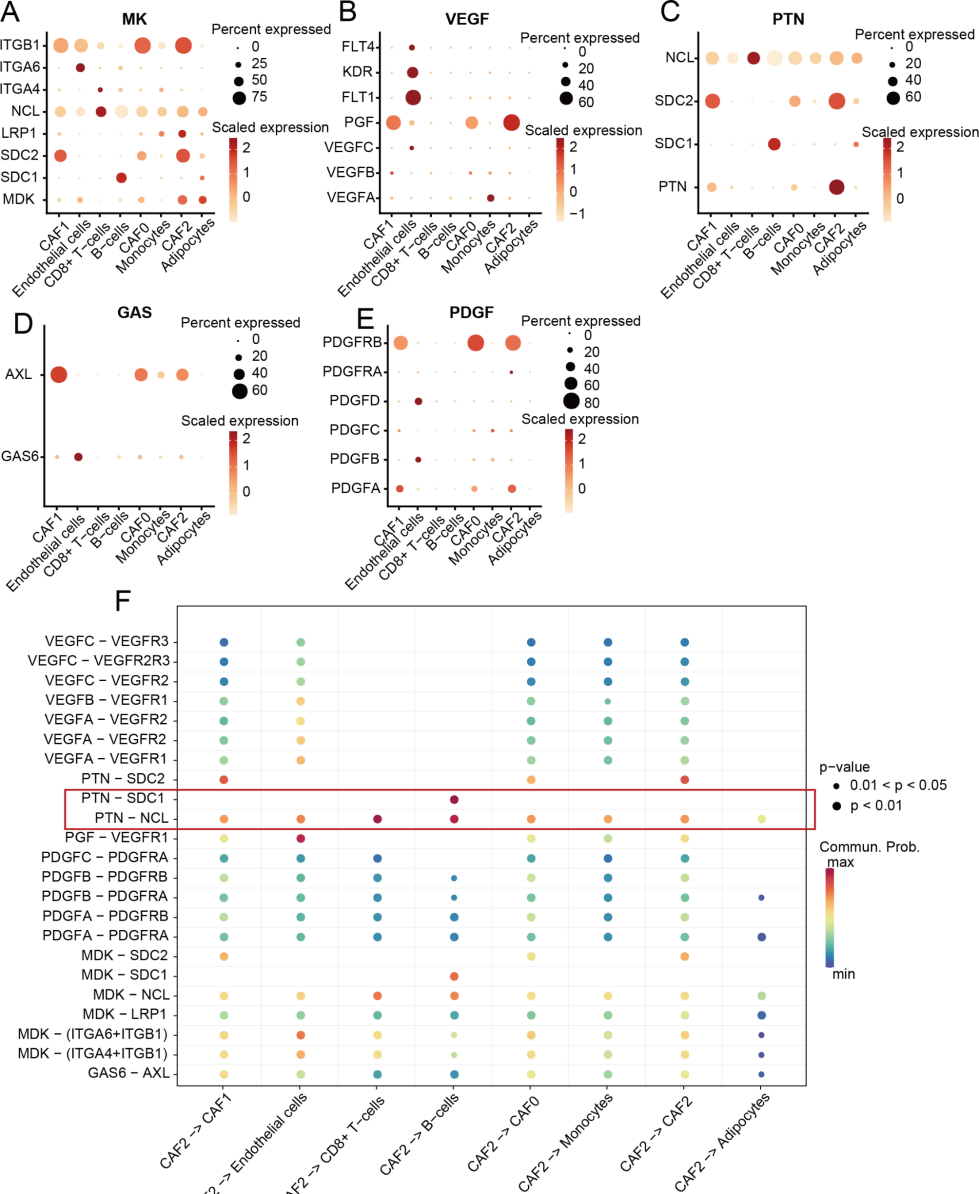
Analysis of the key marker genes among the top five CAF2-related signaling pathways. (**A-E**) The average expression levels of marker genes of the top five CAF2-related signaling pathways among the six cell subsets. (**F**) The contribution of marker genes of the top five CAF2-related signaling pathways to the communication among six cell subsets

Since dysregulation of gene expression was considered to be the origin of CAFs, we calculated the average expression levels of marker genes of top five CAF2-related signaling pathways in cell subsets (Fig. 5A-E). The expression of *PTN*, *SDC1*, and *NCL* was highest in CAF2, B cells, and CD8 + T cells, respectively (Fig. 5C), which implied that *PTN* and *SDC1* played an important role in the communication between CAF2 and B cells. *SDC2* was also highly expressed on CAF1 and CAF0, suggesting potential as a key marker gene shared among CAF0-2 subclasses (Fig. 5C). Subsequently, we analyzed the contribution of marker genes interaction of top five CAF2-related signaling pathways to the communication between cell subsets. The results revealed that the interaction of *PTN-SDC1* and *PTN*-*NCL* contributed the most to the CAF2-B cells interaction, and the interaction of *PTN*-*NCL* showing the most weight intensity on the interaction of CAF2-CD8 + T cells (Fig. 5F). Moreover, the interaction of *PTN*-*NCL* contributed to almost every communicated interaction between cell subsets (Fig. 5F). Therefore, the *PTN*-*SDC1* and *PTN*-*NCL* interactions may regulate cancer-associated fibroblast in HCC by modulating B cells and CD8 + T cells, and *PTN* may be a novel mediator of CAF in HCC.

### Expression of ***PTN*** among NAFLD, cirrhosis, and HCC

**Fig. 6:**
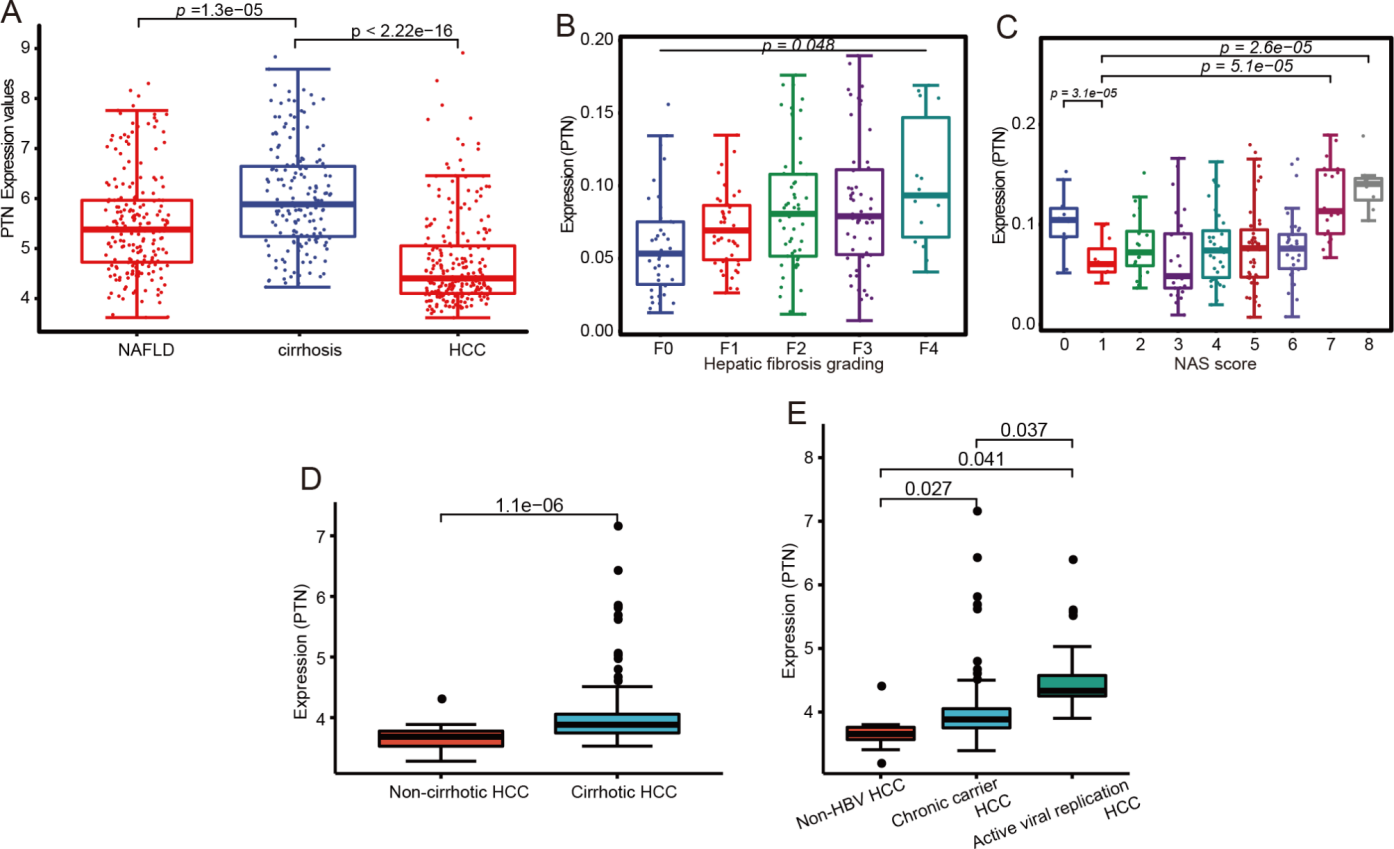
The expression of *PTN* in different tissues and histopathological stages. (**A**) The differential expression of *PTN* among NAFLD, cirrhosis, and HCC. (**B**) Expression of *PTN* among different stages of cirrhosis. (**C**) The expression level of *PTN* in the NAFLD groups based on NAS score. (**D**) The differential expression level of *PTN* between cirrhotic HCC and non-cirrhotic HCC. (**E**) The differential expression level of *PTN* among non-HBV HCC, chronic carrier HCC, and active viral replication chronic carrier HCC.

Liver fibrosis/cirrhosis is an important pathological change in the early stage of HCC. As hallmark features of TME in HCC, we postulated that *PTN* may mediate the role of CAF in cirrhosis-HCC progression. Hence, we compared the *PTN* expression level among NAFLD, cirrhosis, and HCC. The results showed that *PTN* was highly expressed in cirrhosis compared with NAFLD and HCC (Fig. 6A), suggesting its role in initiating liver fibrosis/cirrhosis. Additionally, the expression level of *PTN* showed an increasing trend in liver cirrhosis samples with disease stage (F0-F4) (Fig. 6B). In the NAFLD grouping based on NAS score, the expression level of *PTN* in the samples with NAS score = 7 and 8 was significantly higher than that in the other groups (Fig. 6C). In addition, we found that *PTN* was significantly higher in cirrhotic HCC than in non-cirrhotic HCC (Fig. 6D). Moreover, *PTN* was highly expressed in active viral replication chronic carrier HCC, followed by chronic carrier (CC) HCC when compared with non-HBV HCC (Fig. 6E). These results further suggested *PTN* as possible a novel mediator of CAF in HCC, especially for HBV related cirrhosis-HCC progression. Subsequently, we investigated the relationship between *PTN* expression and immune of TME, and found a significant correlation between the expression of *PTN* and the fraction of 11 cells. In particular, *PTN* had the most significant association with B cells memory and T cells CD8 (Figure S6), which further confirmed *PTN*’s role in mediation of B cells and CD8 + T cells in the cancer-associated fibroblast-related HCC.

### Verification of the modulatory role of ***PTN*** in HBV cirrhosis-HCC progression

**Fig. 7:**
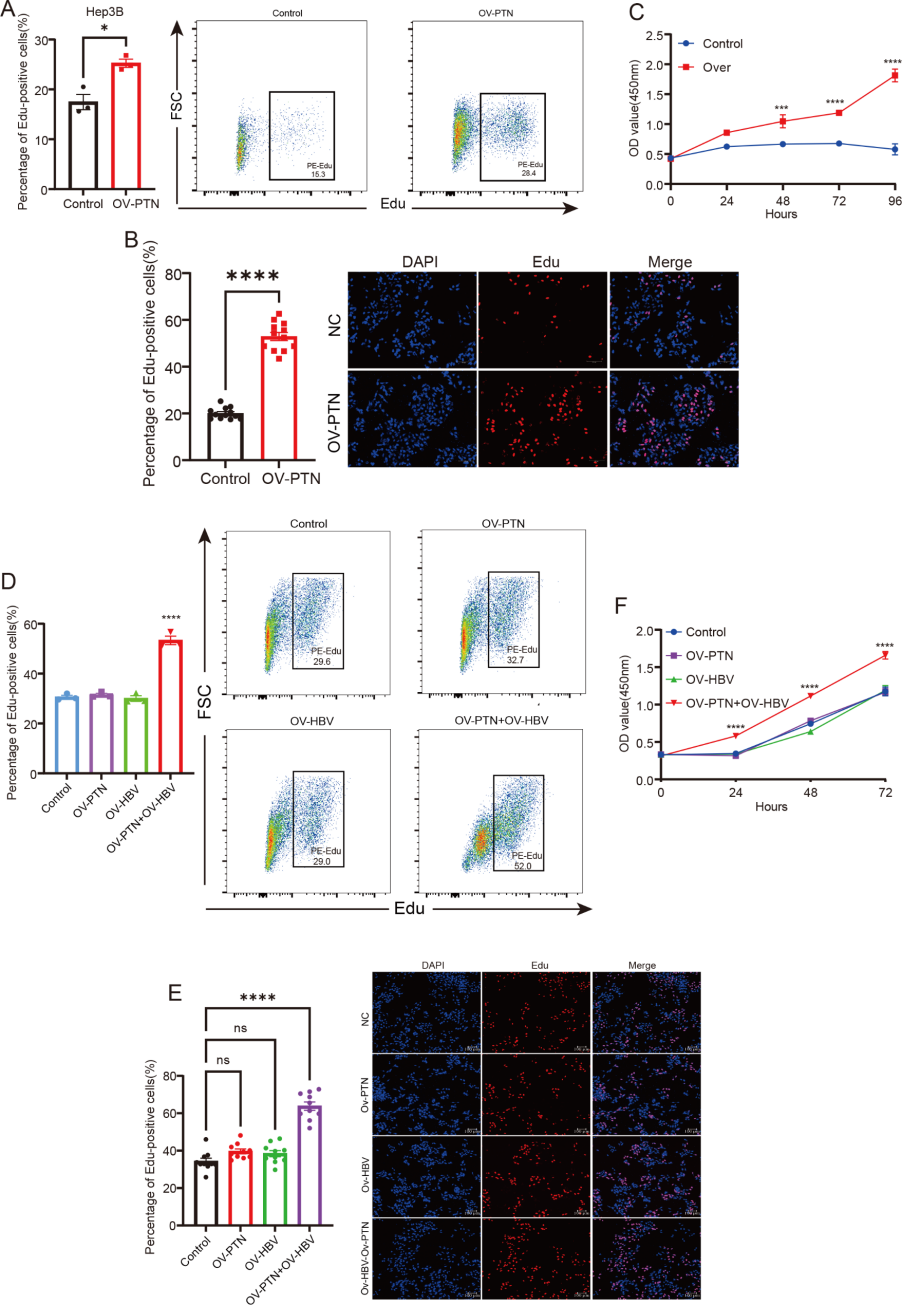
The effect of *PTN* on hepatocarcinogenesis, and cirrhosis-HCC progression following HBV infection. The effect of *PTN* overexpression on proliferation of Hep3B cells as determined by (**A**) FCM, (**B**) IF, and (**C**) CCK-8. The proliferation of Huh7 cells transfected with pHBV1.3 or with *PTN* overexpression or normal plasmid as determined by (**D**) FCM, (**E**) IF, and (**F**) CCK-8.

Since HBV infection is an important cause of HCC and liver fibrosis/cirrhosis is the key intermediate link of HBV-HCC, we explored whether *PTN* can mediate the role of CAF in cirrhosis-HCC progression. Thus, we further investigated whether HBV infection affected the mediatory role of *PTN* on cancer-associated fibroblast-related HCC. Using plasmid pcDNA3.1-*PTN* used for overexpression of *PTN*, we verified *PTN* overexpression using qPCR and WB both in Hep3B and Huh7 cell lines (Figure S7A, Figure S8A). Hep3B was first marked with EDU and then was quantified using FCM and IF, which showed that the activity of cell in the overexpression of *PTN* was significantly increased (Fig. 7A, B). The CCK-8 (Fig. 7C), transwell assays (Figure S7B), and wound healing assay (Figure S7C) showed that the overexpression of *PTN* promoted the Hep3B cell line proliferation, invasion, and activity. However, we could not observe the distinct difference among these methods, including FCM (Figure S8B), CCK-8 (Figure S8C), wound healing assay (Figure S8D), transwell assays (Figure S8E) and IF (Figure S8F), when the overexpression of *PTN* was conducted in the Huh7 cell lines.

Since Hep3B was HBV-positive cell whereas Huh7 was HBV-negative cell, we hypothesized that HBV infection may play an important role in initiating PTN-mediated tumor-associated fibrosis in HCC. Therefore, the Huh7 was transfected with pHBV1.3 containing complete HBV genome to simulate HBV-positive cell. As expected, the simultaneous transfection with plasmid pcDNA3.1-PTN and HBV significantly promoted the cell activity, whereas the effect was weakened when transfection with pcDNA3.1-PTN and HBV alone ((Fig. 7D, E). In addition, the CCK-8 (Fig. 7F), transwell assays (Figure S9A), and wound healing assay (Figure S9B) yielded similar results. These results provided evidence for *PTN’*s role as a novel mediator of cancer-associated fibroblast in HBV infection-caused cirrhosis-HCC progression.

## Discussion

The pathogenesis of HCC is closely related to liver fibrosis caused by chronic hepatitis virus infection. Therefore, HCC mostly co-occurs with liver fibrosis or cirrhosis, suggesting that hepatic fibrosis plays an important role in the formation of precancerous lesions of the liver [15]. CAFs is considered one of the most abundant stromal cells in HCC, that produce large amounts of collagens, leading to the formation of fibrosis and cancer development [16]. Hence, CAF particates in the pathogenesis of HCC, especially HBV cirrhosis-HCC progression, and can thus be an ideal target for the design of agents for inhibiting liver fibrosis [7]. We exploited the precision of single-cell sequencing in determining the cellular and molecular heterogeneity of tumor samples and integrated bioinformatics analyses to study the molecular typing, clinical features, cell communication, and signaling pathways of CAF in HCC. The study found and verified that *PTN* was an important biomarker of CAF in HCC and could be an significant mediator for HBV cirrhosis-HCC progression.

With the progress of HCC treatment technology, much progress has been made in the clinical management of HCC. In particular, the recent five years of clinical application of targeted drugs for HCC have brought huge benefits to patients [4]. However, the five-year survival rate of HCC has not been substantially improved. Factors such as tumor metastasis, recurrence and drug resistance of targeted drugs are obstacles limiting the further improvement of the diagnosis and treatment level of HCC [4, 17]. Therefore, early detection, early diagnosis and early surgical resection are the most important methods to effectively improve the quality of life of patients [17]. Thus, the discovery of biomarkers for the early diagnosis of HCC is still the focus of researchers [18]. The HBV infection is the starting factor, HCC is the final result, and liver cirrhosis is the intermediate stage. Liver cirrhosis plays a bridging role in HBV fibrosis -HCC progression [15]. Inhibiting HBV invasion and reducing the progression of liver cirrhosis may be an important means to prevent the occurrence of HCC during HBV infection [19]. However, HBV fibrosis/cirrhosis of HCC progression lacks specific biomarkers [18]. The *PTN* found in this study may be a CAF specific biomarker and an important molecular mediator mediating HBV infection-related liver fibrosis/ cirrhosis and HCC. To further study on *PTN*, specially, the research of liquid biopsy technology based on *PTN* is helpful to improve the diagnosis and treatment level of HCC.

Different from second-generation sequencing, here, based on single-cell sequencing data, we used Cell cluster analysis and Cell type annotation methods to accurately mine high-purity fibroblast cells and target genes. Single-cell sequencing data can be used to infer and discover cell types in an unbiased manner, while analyzing the genome, transcriptome, and epigenome of individual cells. Therefore, we obtained fibroblasts with subset 2 and 8. The trajectory analysis further clarified that fibroblasts appear early in HCC, indicating the role of fibroblasts in HCC pathogenesis. Then, the correlation analysis between fibroblasts and second-generation sequencing clinical information was carried out using multi-system biostatistics method, which could not only overcome the small number of single cell samples and the lack of clinical data, but also avoid the deviation of second-generation sequencing data. Finally, we use Cell communication analysis based on CellChat algorithm to carry out cell communication. cells communicated weight analysis of signaling pathways, the target gene *PTN* of CAF and B cells and CD8 + T cells mediating cirrhosis-HCC progression were obtained. The precise inference, systematic quantification and comparative analysis of complex cell communication networks by CellChat algorithm can be used to investigate cell communication from different molecular levels [12]. Thus, these analyses focused on cell population-fibroblast -CAF-*PTN*, which was a layer by layer analysis process of tissues-cells-genes. The above research methods provided support for excavating the regulatory genes of cirrhosis-HCC progression. However, some limitations should be identified. First, the research method lacked comprehensive verification and needed more data support. Secondly and inevitably, the method of this study was non-specific, incomplete and universal, which required the verification of biomarkers in cell and animal experiments. Thirdly, the role of *PTN* in mediating CAF in cirrhosis-HCC progression need further exploration.Data has shown that CAFs regulate many wide-range of genes and transcription factors to activate signals which trigger fibroblasts to promote carcinogenesis [16]. Previous study showed that the genes upregulated in tumor subtypes were also prominently expressed in fibroblasts, which is the main cell type in the stroma [20]. In this study, we found novel CAFs-derived gene signatures that play an important role in the development of HCC. We found that *PTN* was a novel mediator of CAF in HBV cirrhosis-HCC progression. PTN (encoding by the *PTN* gene) is a small cationic protein with potent mitogenic and angiogenic activity, and has been associated with a wide range of important biological events, including tumorigenesis [21]. Fibroblast growth factor and signaling proteins are known to increase *PTN* expression level, resulting in activation of fibroblasts which then aggressively promote cancer progression [22, 23]. Thus, *PTN* may be a promising therapeutic target for cancer treatment [24]. Previous research showed that *PTN* enhances the growth of hepatocytes and its expression is increased after severe liver injury, such as partial hepatectomy and hepatitis, and promotes liver regeneration [25]. However, *PTN* has been associated with hepatic fibrosis and hepatocarcinogenesis. For example, Kohashi et al. found that *PTN* expression was increased in carbon tetrachloride-induced fibrotic liver [26]; Park et al. found that *PTN* was expressed in HSCs, Kupffer cells, and hepatocytes from fibrotic liver. It inhibited the TGFb1-induced apoptosis thereby promoting liver fibrogenesis and carcinogenesis [27]. In this study, we uncovered *PTN* to be an important mediator of HBV cirrhosis-HCC progression through integrated bioinformatics analysis and validated its clinical significance in cirrhosis-HCC progression patients. Further investigations into the role of *PTN* in liver fibrogenesis and carcinogenesis may lead to the development of *PTN-*targeted therapies for HCC.

Here, we found strong interaction of CAF2 with B cells and CD8 + T cells, suggesting that CAF may contribute to the remodeling of immune microenvironment and the interaction of CAF2-B cells and CAF2-CD8 + T cells may mediate the regulatory role of *PTN*. Close functional links between inflammation and fibrosis have been reported, which involve several key players such as B cells and T cells that contribute to liver fibrosis and HCC [28]. Several proinflammatory mediators, including IL-1, IL-6, and TNF-α are derived from inflammatory cells such as B cells and T cells within the chronically injured liver and can contribute to hepatocarcinogenesis [29]. These findings are consistent with our results that the ability of CAFs to modulate the immune system and the interaction between CAF2-B cells and CAF2-CD8 + T cells is mediated by *PTN.* [30] Analysis of the relationship between *PTN* expression and immune of TME uncovered *PTN* was associated with B cells memory and CD8 + T cells. Though CD8 + T cells contributed to anti-tumor responses, previous research found that T cells are mainly restricted to stromal zones, including fibroblasts, and CAFs may induce antigen-specific deletion of CD8 + T Cells to protect tumor cells [31]. Goplen et al. revealed CD8 + T cells accumulate in the lungs did not have the ability to fight infection, but are involved in the development and maintenance of lung inflammation and fibrosis [32]. We speculated that T cells CD8 + lost its protective properties in the microenvironment of liver fibrosis, or loses its anti-fibrosis function due to depletion. We also found that the interaction of *PTN*- *SDC1* and *PTN*- *NCL* may mediate the effect of B cells and CD8 + T cells on cancer-associated fibroblast-related HCC. *PTN* have been implicated in numerous inflammatory conditions such as chronic hepatitis. Hepatic stellate cells regulate the hepatic immune cells such as macrophages, B cells, T cells, and natural killer cell, by releasing a wide variety of chemokines to promote fibrogenesis [33, 34]. Thus, *PTN* may be involved in the regulation of hepatic immune system by modulating hepatic stellate cells, although further research is necessary to validate this phenomenon.

HBV infection has been linked to the development of liver fibrosis and HCC [5]. In our study, we found that *PTN* derived from CAF had a much greater effect on HBV-related HCC than non-HBV-related HCC. Transfection of HBV plasmid into non-HBV-related HCC cells significantly promoted the activity of *PTN* in tumor cells. Clinical analysis also revealed that *PTN* was associated with liver cirrhosis and HCC, which further indicated that *PTN* may be an important target of cirrhosis and HCC induced by HBV. Although *PTN* has been reported to play a role in fibrogenesis [26] and tumorigenesis [21], the role of *PTN* in hepatitis B virus cirrhosis-HCC progression has not been sufficiently studied. *PTN* is regulated by miRNA and hepatitis B virus X protein is associated with the pathogenesis of HBV-related HCC [35, 36]. However, few studies have explored the mechanism by which *PTN* contribute to fibrosis in HCC. The aim of our research was to identify novel biomarkers associated with fibrosis/cirrhosis-HCC progression due to HBV infection. Since *PTN* was previously linked to the occurrence of liver fibrogenesis [26] and tumor development [21], we hypothesized that *PTN* may promote the progression of liver fibrosis/cirrhosis to HCC. Therefore, integrated analysis of big data from clinical samples was conducted to explore the clinical value of *PTN* in cirrhotic and HCC. Collectively, we found that *PTN* derived from CAFs acted as a novel mediator of the CAFs’s effects on HBV cirrhosis-HCC progression.

In conclusion, this study found that *PTN* derived from CAFs participated in hepatocarcinogenesis by regulating fibrosis, and that *PTN* may mediated the effects of CAF on HBV infection associated with liver fibrosis in HCC. These findings suggest the role of *PTN* in HBV fibrosis/cirrhosis-HCC progression and its value into the clinical management of HBV fibrosis and HCC.

## Electronic supplementary material

Below is the link to the electronic supplementary material.

**Figure S1**. The appropriate amount of principal component analysis (PCA) was assessed for further dimension reduction. (A) Analysis of the importance of the first 20 PCA. The key PC is shown above the dotted line and has a lower *P* value. (B) The lithotripsy diagram showing the standard error of each PCA. The standard error was used to explain data variance

**Figure S2**. Single cell clustering results for the GSE151530 dataset. (A) Analysis of the importance of the first 20 PCA in the GSE151530 dataset. The key PC is shown above the dotted line and has a lower P value. (B) The lithotripsy diagram showing the standard error of each PCA in the GSE151530 dataset. The error is mainly used to explain the data variance. (C) UMAP showing the annotation for different groups of cell types. (D) UMAP representing three subclasses of fibroblasts cells. (E) Dot plot illustrating the top 10% of differential marker genes from 3 subclasses of the GSE151530 dataset. The overlapping genes among the three subclasses of fibroblasts cells between GSE125449 and GSE151530 in (F) sub-cluster0, (G) sub-cluster1, and (H) sub-cluster2

**Figure S3**. The relationship between CAF2 scores and different clinical features. The association between CAF2 score and (A) tissue type, (B) sex, and (C) age of HCC patients

**Figure S4**. The relationship between CAF0, 1 score, and different clinical features. The association between CAF0 score and (A) HCC pathological grade, (B) tissue samples, (C) pathological grade, (D) tissue type, (E) sex, and (F) age of HCC patients. The association between CAF0 score and (G) HCC pathological grade, (H) tissue samples, (I) pathological grade, (G) tissue type, (H) sex, and (L) age of HCC patients. Mixed: mixed sample

**Figure S5**. The communication links between CAF0-2 and different cell subsets. The associatin network of (A) CAF1, (B) endothelial cells, (C) CD8 + T − cells, (D) B − cells, (E) CAF0, (F) monocytes, (G) CAF2, (H) adipocytes with different cell subsets

**Figure S6**. Correlation matrix between the immune cell fraction and *PTN*. The ovals represent significantly correlated relationships, the red ovals are positively correlated and the blue ovals are negatively correlated, and the staining density indicate the correlation coefficient size

**Figure S7**. The role of *PTN* in hepatocarcinogenesis of Hep3B cell line. (A) The overexpression of *PTN* was verified through qPCR and WB in Hep3B cell line. (B) Transwell assays and (C) wound healing assay were conducted to measure the cell invasion and migration activities, respectively, following transfection with *PTN* overexpression and normal control

**Figure S8**. The effect of *PTN* on hepatocarcinogenesis of Huh7 cell lines. (A) The overexpression of *PTN* was verified through qPCR and WB in Huh7 cell lines. The proliferation of Huh7 cells carrying *PTN* overexpression or normal as determined by (B) FCM, (C) CCK-8. Cell invasion and migration activities as measured by (D) wound healing assay and (E) Transwell assays, respectively. The proliferation of Huh7 cells carrying *PTN* overexpression or normal as determined by (F) IF.

**Figure S9**. Verification the effect of *PTN* in the HBV infection associated with the cirrhosis-HCC progression. The cell invasion and migration activities as determined by (A) Tanswell assays and (B) wound healing assay, respectively, in Huh7 cells transfected with pHBV1.3 or with *PTN* overexpression or normal expression

Supplementary Material 10

## Data Availability

Data and material are provided along with the manuscript.

## Abbreviations

CAFs: Cancer-associated fibroblasts
HCCs: Hepatocellular carcinomas
HBV: Hepatitis B virus cirrhosis
CAF0-2: Three cell subclasses
TME: Tumor microenvironment
NAFLD: Non-alcoholic fatty liver disease
PCA: Principal component analysis
qPCR: Real-time quantitative PCR
CCK-8: Cell Counting Kit-8
FCM: Flow cytometry
IF: Immunofluorescence
