# Supplementary figures and images for: Single-cell RNA sequencing reveals the mediatory role of cancer-associated fibroblast PTN in hepatitis B virus cirrhosis-HCC progression
Source: Gut Pathog. 2023 May 31;15:26. doi: 10.1186/s13099-023-00554-z (PMC10230711; doi:10.1186/s13099-023-00554-z)

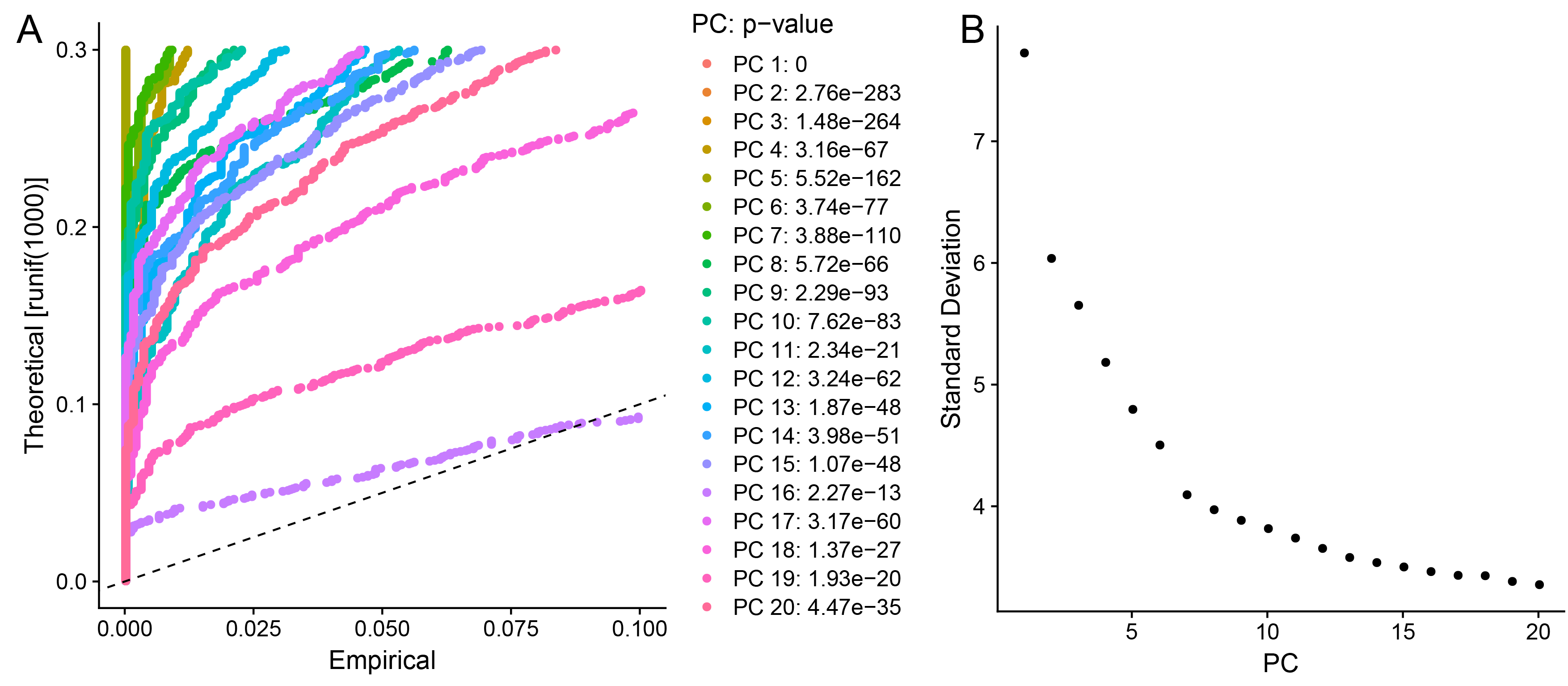

Supplement: Supplementary file 1 — Figure S1. The appropriate amount of principal component analysis (PCA) was assessed for further dimension reduction. (A) Analysis of the importance of the first 20 PCA. The key PC is shown above the dotted line and has a lower P value. (B) The lithotripsy diagram showing the standard error of each PCA. The standard error was used to explain data variance [file 13099_2023_554_MOESM1_ESM.tif]

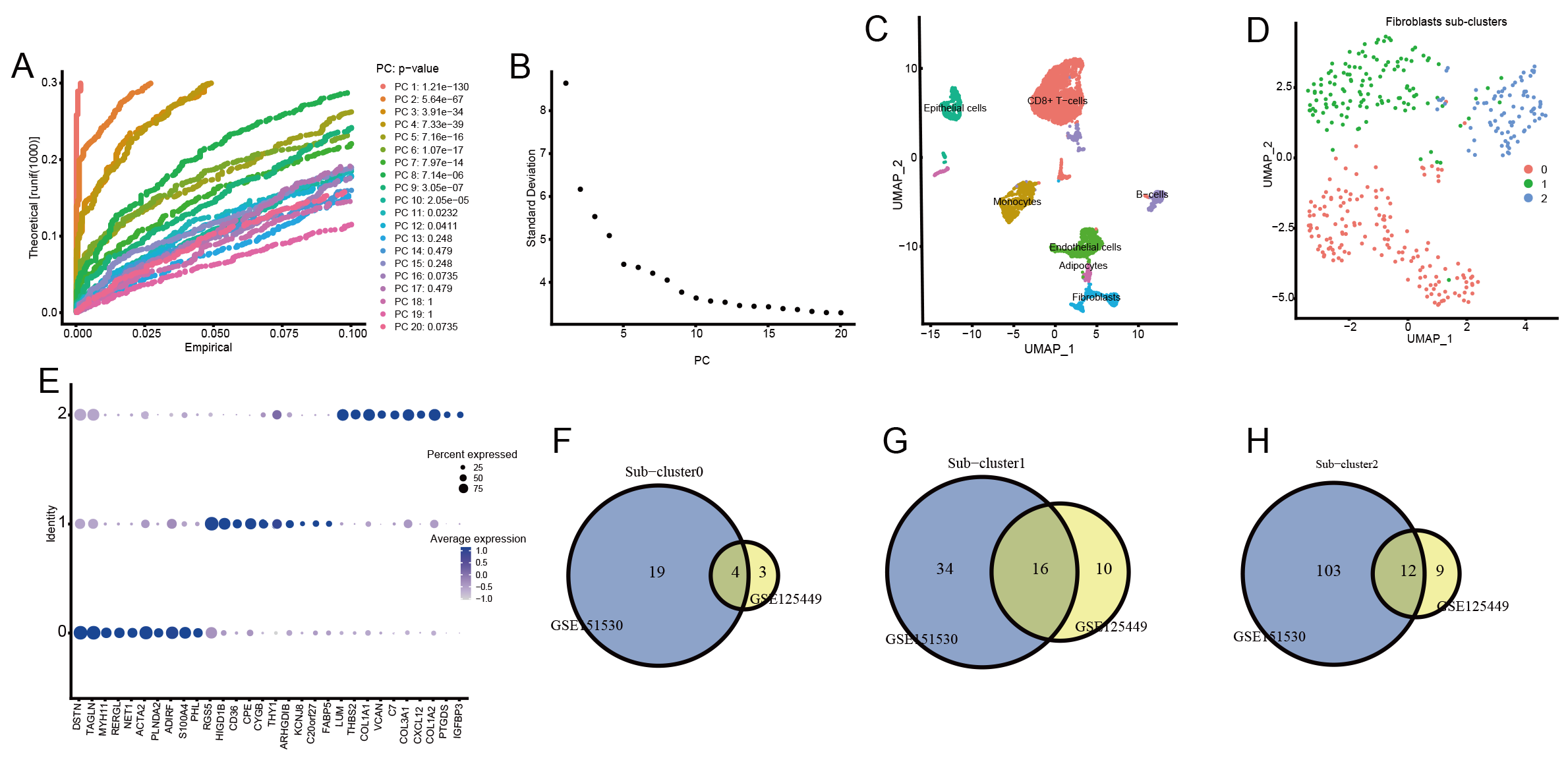

Supplement: Supplementary file 2 — Figure S2. Single cell clustering results for the GSE151530 dataset. (A) Analysis of the importance of the first 20 PCA in the GSE151530 dataset. The key PC is shown above the dotted line and has a lower P value. (B) The lithotripsy diagram showing the standard error of each PCA in the GSE151530 dataset. The error is mainly used to explain the data variance. (C) UMAP showing the annotation for different groups of cell types. (D) UMAP representing three subclasses of fibroblasts cells. (E) Dot plot illustrating the top 10% of differential marker genes from 3 subclasses of the GSE151530 dataset. The overlapping genes among the three subclasses of fibroblasts cells between GSE125449 and GSE151530 in (F) sub-cluster0, (G) sub-cluster1, and (H) sub-cluster2 [file 13099_2023_554_MOESM2_ESM.tif]

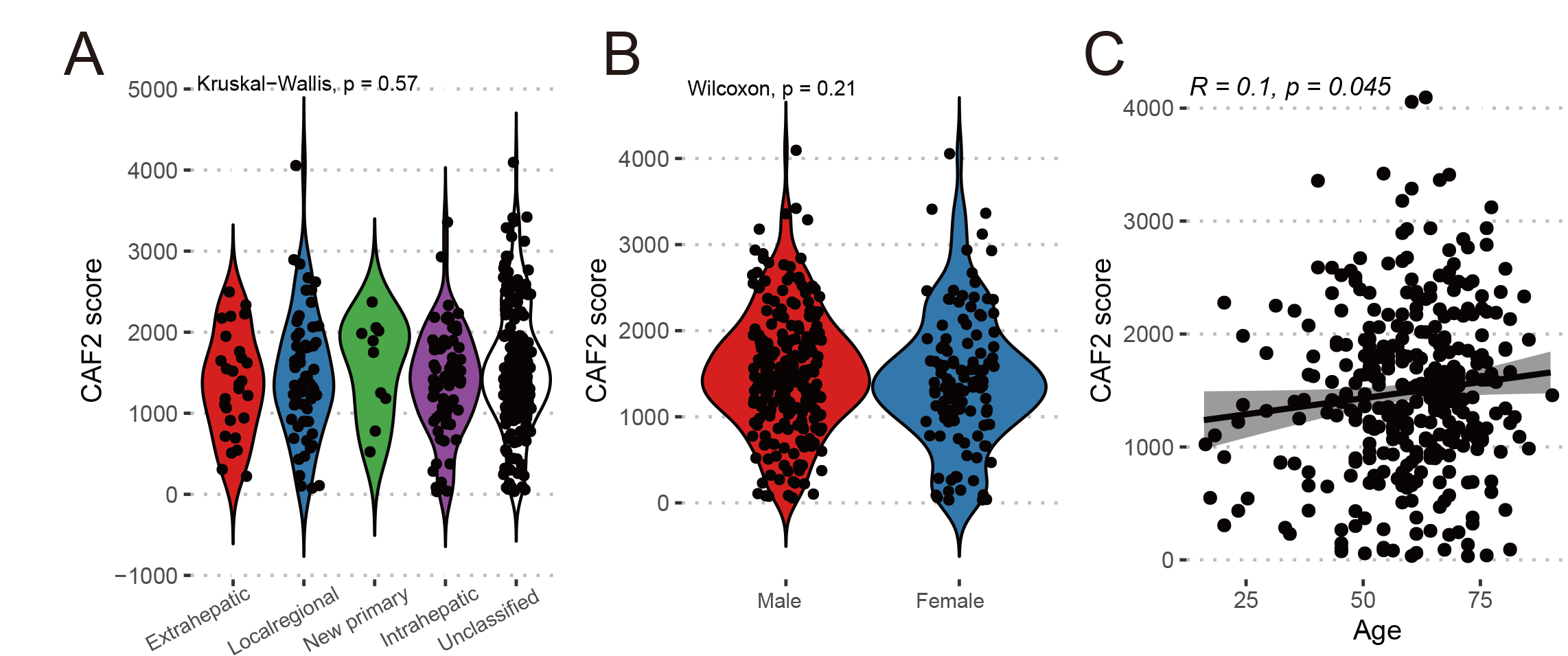

Supplement: Supplementary file 3 — Figure S3. The relationship between CAF2 scores and different clinical features. The association between CAF2 score and (A) tissue type, (B) sex, and (C) age of HCC patients [file 13099_2023_554_MOESM3_ESM.tif]

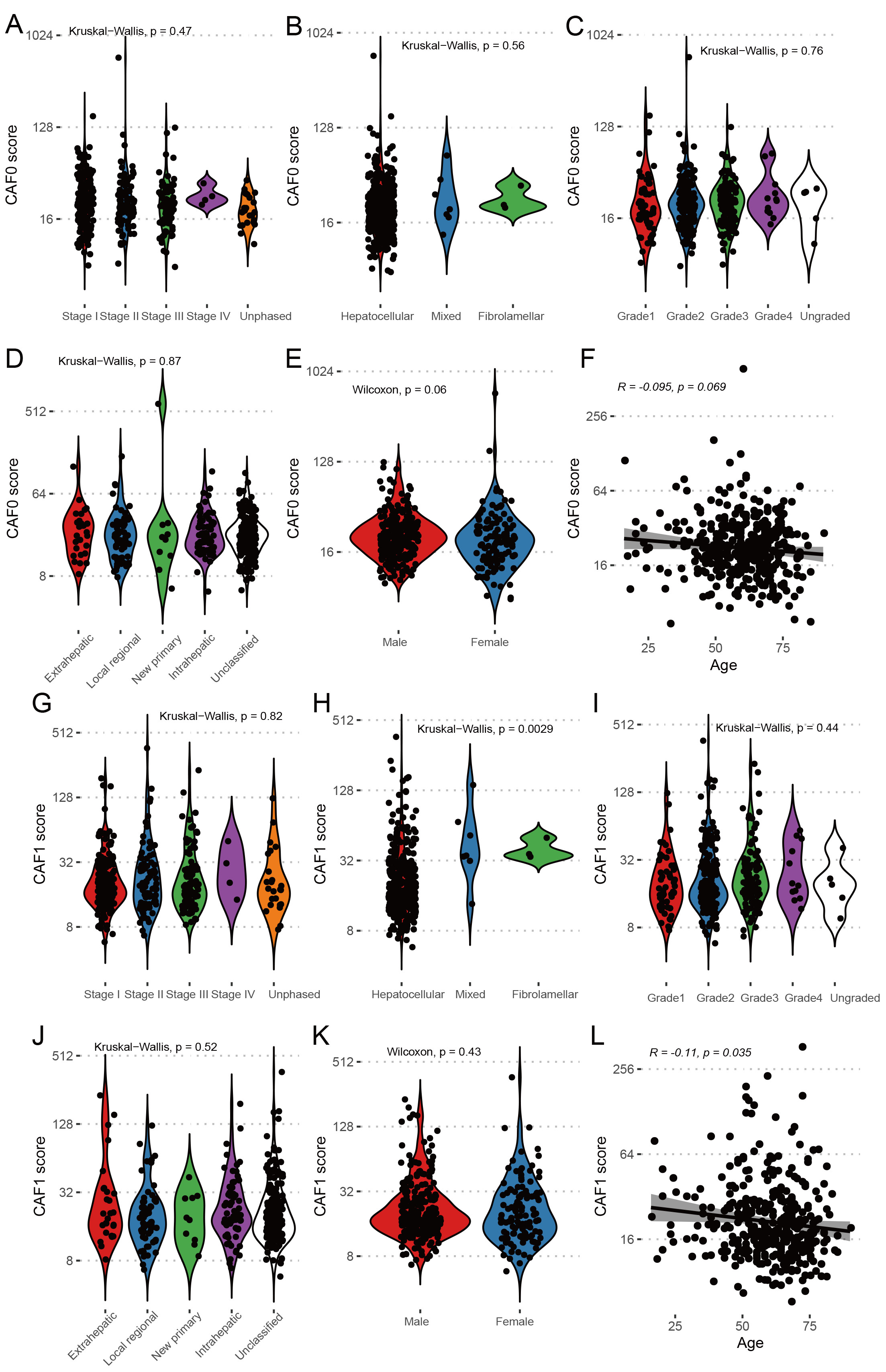

Supplement: Supplementary file 4 — Figure S4. The relationship between CAF0, 1 score, and different clinical features. The association between CAF0 score and (A) HCC pathological grade, (B) tissue samples, (C) pathological grade, (D) tissue type, (E) sex, and (F) age of HCC patients. The association between CAF0 score and (G) HCC pathological grade, (H) tissue samples, (I) pathological grade, (G) tissue type, (H) sex, and (L) age of HCC patients. Mixed: mixed sample [file 13099_2023_554_MOESM4_ESM.tif]

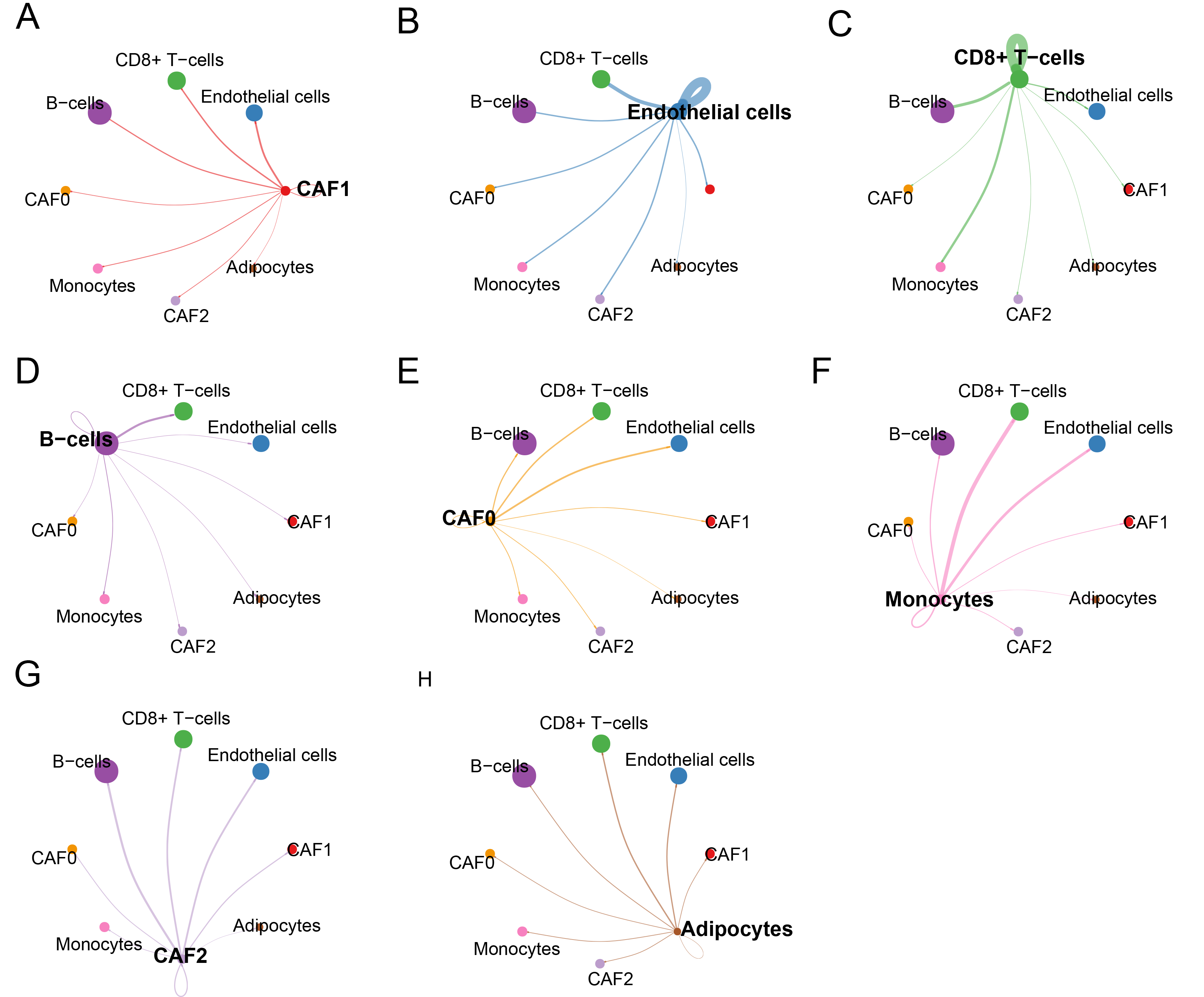

Supplement: Supplementary file 5 — Figure S5. The communication links between CAF0-2 and different cell subsets. The associatin network of (A) CAF1, (B) endothelial cells, (C) CD8 + T − cells, (D) B − cells, (E) CAF0, (F) monocytes, (G) CAF2, (H) adipocytes with different cell subsets [file 13099_2023_554_MOESM5_ESM.tif]

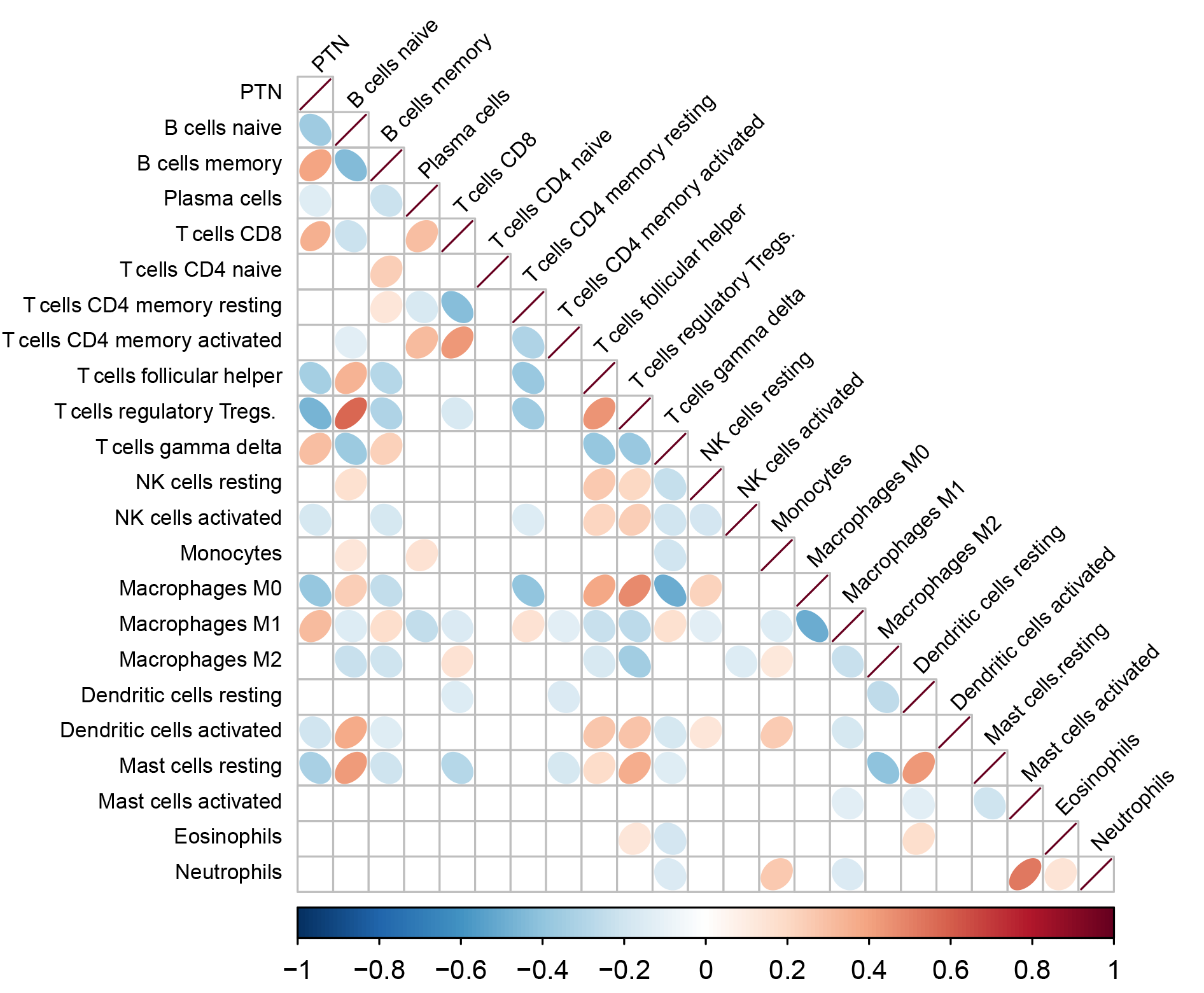

Supplement: Supplementary file 6 — Figure S6. Correlation matrix between the immune cell fraction and PTN. The ovals represent significantly correlated relationships, the red ovals are positively correlated and the blue ovals are negatively correlated, and the staining density indicate the correlation coefficient size [file 13099_2023_554_MOESM6_ESM.tif]

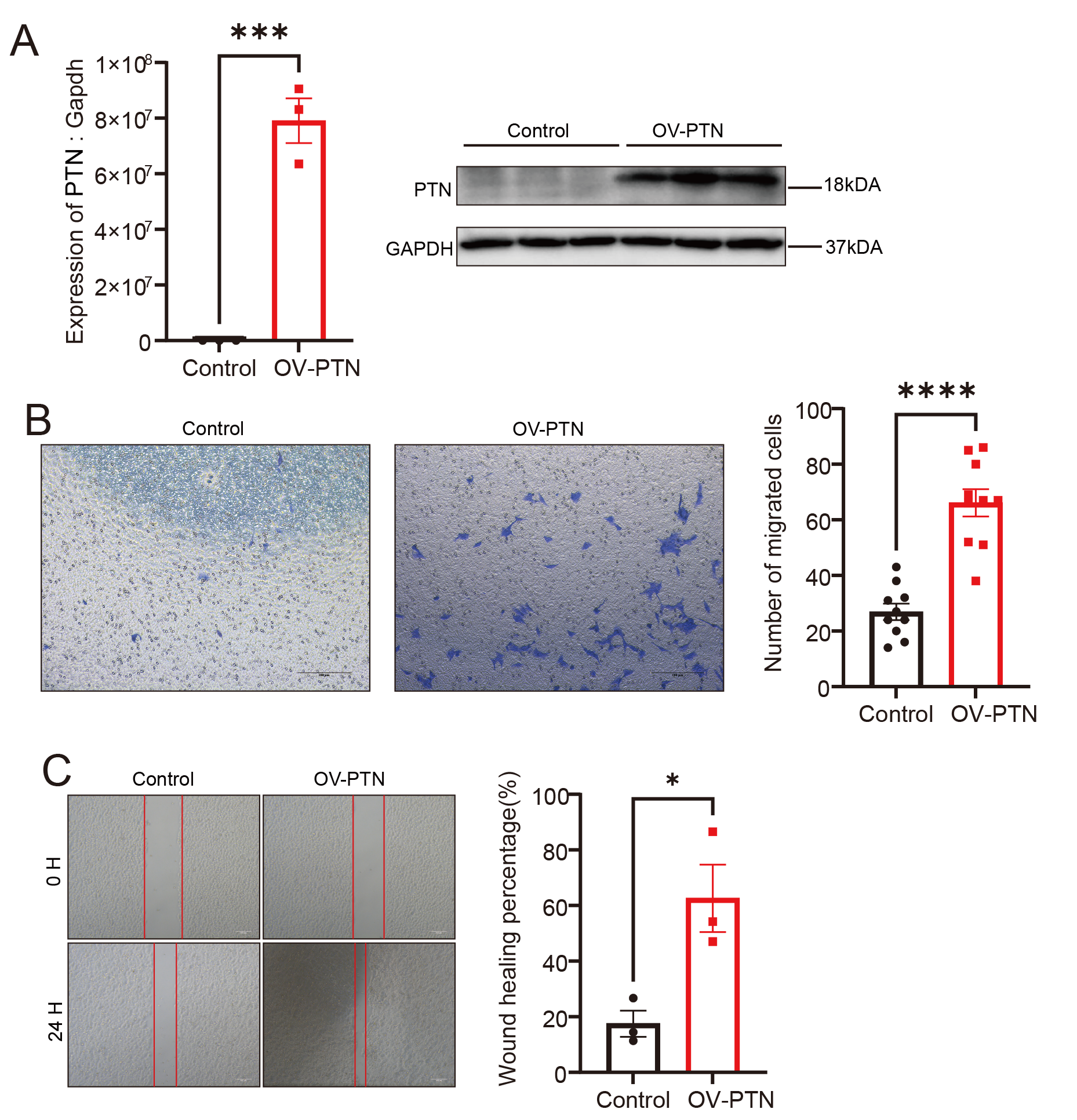

Supplement: Supplementary file 7 — Figure S7. The role of PTN in hepatocarcinogenesis of Hep3B cell line. (A) The overexpression of PTN was verified through qPCR and WB in Hep3B cell line. (B) Transwell assays and (C) wound healing assay were conducted to measure the cell invasion and migration activities, respectively, following transfection with PTN overexpression and normal control [file 13099_2023_554_MOESM7_ESM.tif]

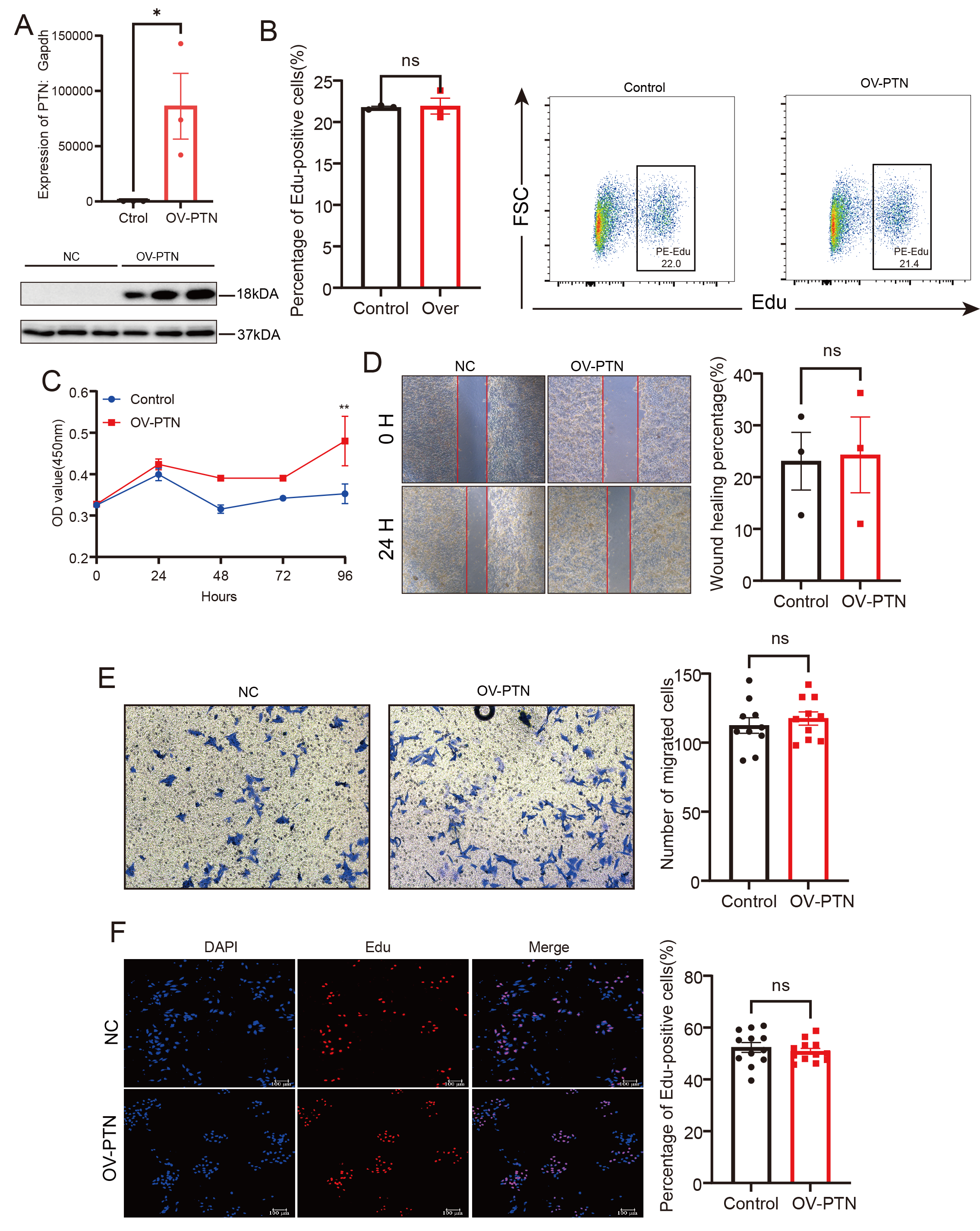

Supplement: Supplementary file 8 — Figure S8. The effect of PTN on hepatocarcinogenesis of Huh7 cell lines. (A) The overexpression of PTN was verified through qPCR and WB in Huh7 cell lines. The proliferation of Huh7 cells carrying PTN overexpression or normal as determined by (B) FCM, (C) CCK-8. Cell invasion and migration activities as measured by (D) wound healing assay and (E) Transwell assays, respectively. The proliferation of Huh7 cells carrying PTN overexpression or normal as determined by (F) IF. [file 13099_2023_554_MOESM8_ESM.tif]

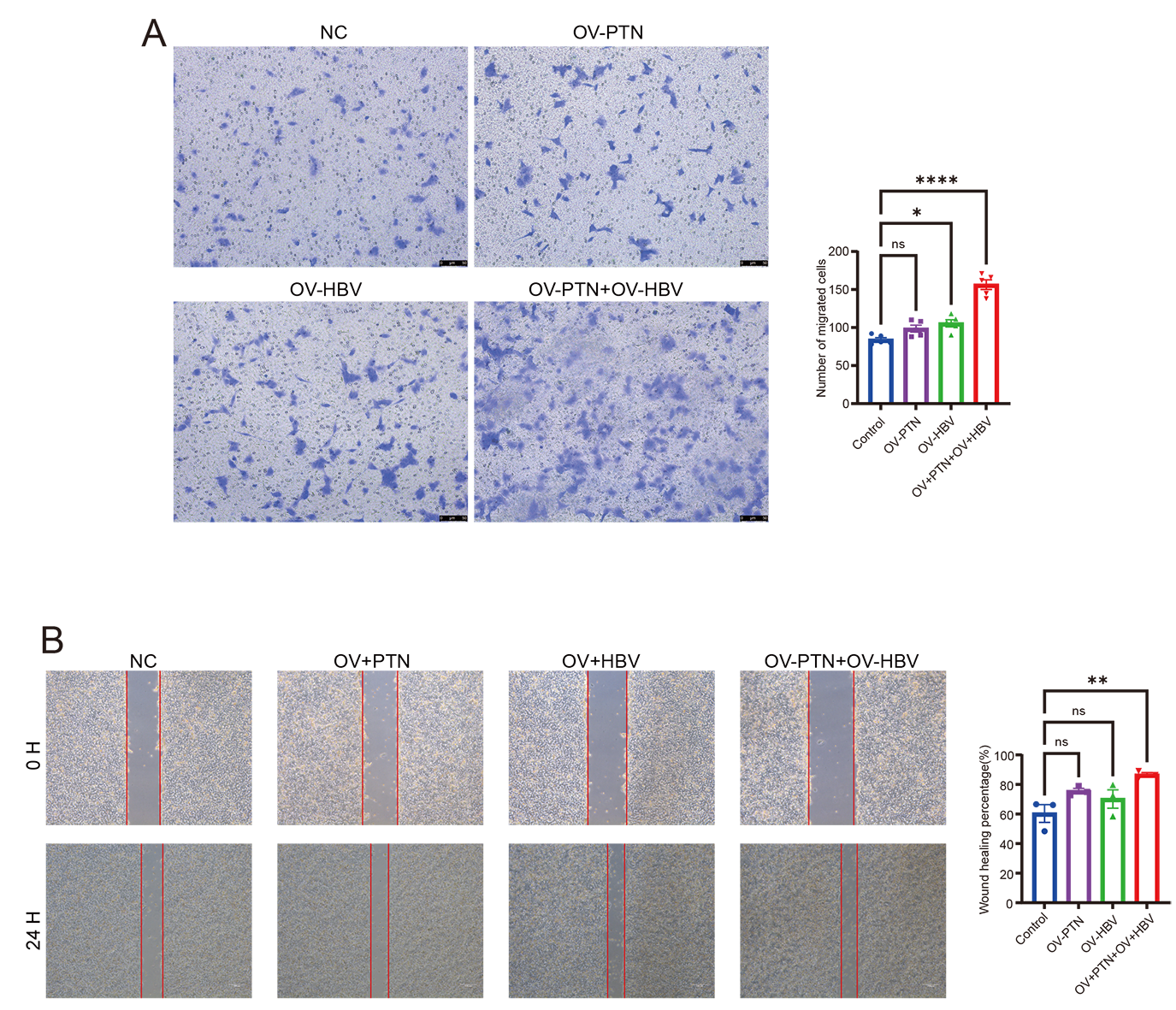

Supplement: Supplementary file 9 — Figure S9. Verification the effect of PTN in the HBV infection associated with the cirrhosis-HCC progression. The cell invasion and migration activities as determined by (A) Tanswell assays and (B) wound healing assay, respectively, in Huh7 cells transfected with pHBV1.3 or with PTN overexpression or normal expression [file 13099_2023_554_MOESM9_ESM.tif]
