# Supplementary material for: Single-cell RNA sequencing reveals the mediatory role of cancer-associated fibroblast PTN in hepatitis B virus cirrhosis-HCC progression
Source: Gut Pathog. 2023 May 31;15:26. doi: 10.1186/s13099-023-00554-z (PMC10230711; doi:10.1186/s13099-023-00554-z)
Supplement: Supplementary file 10 — Supplementary Material 10 [file 13099_2023_554_MOESM10_ESM.docx]

| **Table S1.** **The primers sequences for RT-qPCR in this study.** | | |
| --- | --- | --- |
| **Gene** | **LEFT** | **RIGHT** |
| **GAPDH** | CGGATTTGGTCGTATTGGG | CTGGAAGATGGTGATGGGATT |
| **PTN** | TGGAGAATGGCAGTGGAGTG | GGCTTGGAGATGGTGACAGT |
